# A *modest proposal* to the transplant publik to prevent harm to people with acute myeloid leukaemia in 1st complete remission cured by chemotherapy

**DOI:** 10.1038/s41375-024-02214-w

**Published:** 2024-03-08

**Authors:** R. P. Gale, G. L. Phillips, H. M. Lazarus

**Affiliations:** 1 Centre for Haematology, Department of Immunology and Inflammation, Imperial College of Science, Technology and Medicine, London, SW7 2AS UK; 2 Wake Forest School of Medicine (Emeritus), Winston-Salem, NC USA; 3 Department of Medicine, Division of Hematology and Oncology, Case Western Reserve University, Cleveland, OH USA

**Keywords:** Cancer, Cell biology

*The tiny Lilliputians surmise that Gulliver’s watch* [and expert consensus guidelines] *may be his God, because it is that which, he admits, he seldom does anything without consulting*. Jonathan Swift, *Travels into Several Remote Nations of the World by Lemuel Gulliver* (Gulliver’s Travels) 1726 [1].

Transplants cure some people with acute myeloid leukaemia in 1st complete remission. However, some of these people were already cured by chemotherapy. In these persons a transplant cannot be of benefit but has the potential to be harmful.

The challenge, of course, is distinguishing persons in 1st complete remission already cured by chemotherapy from those who were not. The usual approach is to consider co-variates *at diagnosis* correlated with failing chemotherapy. There are many risk classification models used for this such as the European LeukaemiaNet (ELN), National Comprehensive Cancer Network (NCCN) and Medical Research Council (MRC) risk classifications [2–4]. However, these models are only modestly accurate with Concordance (C)-statistics of 0.80 at best. Put otherwise, these models when applied to a population at diagnosis will incorrectly predict relapse about 20–30% of the time. It Is, of course, hoped accuracy of these models will improve but this is unlikely to do so substantially.

But there are other important limitations. 1st, these models are neither dynamic nor representative. They are based predominately on data from persons < 60 years receiving cytarabine and daunorubicin induction therapy followed by high-dose cytarabine consolidation. Several fail to account for recent developments such as *FLT3*-inhibitors or venetoclax/azacitidine regimens. But more importantly, they lose accuracy when applied to someone in 1st complete remission for a few months when a transplant is being considered. Much of the prediction accuracy of these models is influenced by the likelihood of achieving a 1st complete remission and of very early release rather than the prognosis of someone in complete remission for a few months when a transplant is being considered. It is also important to distinguish prediction models from risk classifications. In many decision-making contexts classification represents a premature decision combining prediction and decision-making which usurps the decision-maker [5, 6]. For example, people often use risk classification data to assign therapy(ies).

Recently, testing for measurable residual disease (MRD) in persons in histological 1st complete remission has been used to predict relapse probability but again with a C-statistics of < 0.80 consistent and a positive predictive value of only about 70 precent (reviewed in [7, 8]). Importantly, we cannot identify who people with a false-positive MRD-test are. It is hoped relapse prediction accuracy will improve by increasing MRD-test sensitivity, for example by testing for leukaemia stem cells [9, 10]. Although this may happen, focusing on improving MRD-test sensitivity ignores the fundamental problems of Poisson noise (sampling error) and inability to distinguish between leukaemia cells detected in the assay from the rare leukaemia cell(s) with the biological capacity and probabilistic chance to cause relapse in someone’s remaining lifetime [11]. Also, substantial prediction accuracy is lost by reporting results of MRD-testing as positive or negative. So although results or MRD-testing in AML can distinguish cohorts with different relapse probabilities they cannot accurately predict if someone in 1st complete remission will relapse. It is also important to recognise predictions from these models reflect population rather than individual risks. For example, some persons in a high-risk cohort in a risk classification model have a lower risk relapse compared with others in a low-risk cohort in the model [6, 12]. This is because the risk-assignment of the model identifies the average of a cohort, not individual risk. Lastly, although some data suggest a positive MRD-test may predict imminent relapse the median lead time to clinical relapse is about 3 months [13]. There are no convincing data a lead time of this magnitude would result in a better transplant outcome. Much literature of MRD-testing is parisology rather than proficuous in accurately predicting relapse in a person in 1st complete remission.

Another consideration is few if any data from randomised controlled trials prove changing therapy such as doing a transplant because someone has a positive MRD-test improves their outcome. Our interpretation is a positive MRD-test identifies adverse laeukemia biology. The data do not prove residual leukaemia cells detected by the MRD-test cause relapse. Association *versus cause-and effect*. To illustrate this controversy, some people suggest transplants not be done in people with a positive MRD-test because outcomes are poor compared with people with a negative MRD-test [14]. This is, of course, a potentially dangerous argument as people who are MRD-test-positive may get the greatest incremental benefit from a transplant. Knowing which strategy is correct requires a randomised controlled trial (RCT); There are none.

Another strategy to distinguish people who cannot benefit from a transplant from those who might is waiting to see if someone in 1st complete remission relapses. Obviously accuracy is 100%. So why not wait? The prevailing argument is if you wait for relapse it may be too late to cure someone who might have been cured were a transplant done in 1st complete remission. This objection is often linked to the notion someone must be in a 2nd complete remission to benefit from a transplant. We suggest this line of reasoning errs.

Why? Although true people transplanted in 2nd complete remission have better outcomes compared with those transplanted not in 2nd complete remission, this most likely reflects a selection bias [15]. People achieving a 2nd complete remission have the most responsive leukaemia making this a *self-fulfilling prophesy*. Observational transplant datasets such as the Center for Blood and Marrow Research (CIBMTR) and European Bone Marrow Transplant Group (EBMT) lack data on people who relapse but do not advance to a transplant including people failing to achieve a 2nd complete remission refused a transplant, people dying from the attempt to achieve a 2nd complete remission and people who cannot advance to a transplant because of chemotherapy-related adverse events which make them unsuitable to proceed to a transplant (infection, kidney or lung failure *etc*.). This limitation of transplant registries and of reports from transplant centres makes it impossible to draw solid conclusions about the fate of people relapsing from 1st complete remission. But perhaps the most important consideration is there are no data from a RCT indicating a benefit to attempting to achieve a 2nd complete remission before considering a transplant when everyone is considered. In fact, there are recent data to the contrary [16].

Bearing in mind the dictum *primum no nocere* we suggest the best strategy is to wait to see who relapses and use transplants only in them. In those relapsing there are no convincing data one should try to achieve a 2nd remission by giving chemotherapy. There are, of course, some circumstances which may necessitate a delay before proceeding directly to a transplant such as uncontrolled infection or a very high blast concentration but these people may also not be a candidate to receive another course of chemotherapy to achieve a 2nd complete remission. Some of these contra-indications can be reversed by antibiotics, transfusions and cytarabine or hydroxyurea. Many medical decisions are probabilistic and physicians often feel they need to make binary decision at 1 timepoint, in this instance recommend a transplant or not. However, often the best decision is *no decision, get more data*.

What might be objections to our *modest proposal*? Before addressing this we need to consider the concepts of actions, intentions and consequences which fall in the spheres of moral philosophy and ethics. Many readers will be familiar with the runaway trolly dilemma formulated by Foot and modified by Thomson [17, 18]. Imagine you are standing beside trolly tracks and spot a distant runaway trolley speeding towards 5 unaware workers. You notice a lever on the track which, if you throw it, will divert the trolley to a second track where there is only 1 unaware worker. Would you throw the lever resulting in 1 death but saving 5 lives, a net gain of 4 lives? Most people say yes. Now consider the same scenario where you are on a bridge over the tracks. Hurling a man standing beside you onto the tracks will also divert the runaway trolly resulting in 1 death but saving 5 lives, a net gain of 4. Would you do it? Most people say no. Finally, to put these hypotheticals in a medical context imagine you have 5 patients who will die unless they receive an organ transplant immediately. Unfortunately, there are no donors. You also have 1 patient recovering from a myocardial infarction who, if killed, could donate organs to 5 others, again a net gain of 4 lives. Would you kill the recovering patient? Most physicians say no. (This scenario differs from a haematopoietic cell transplant donor who usually experiences no harm.).

Why bother you, the reader, with these hypotheticals. The point, rationality aside, is whether an action is passive (throwing a switch) or active (hurling someone off a bridge) matters. This distinction is between killing someone *versus* letting someone die. The person throwing the lever saves 5 lives at the cost of 1. Throwing the lever does not kill the 1 directly, the runaway trolley is the proximal cause of death. In contrast, in hurling someone off the bridge or in sacrificing your healthy patient you are the proximal cause of death.

This dichotomy in peoples’ perception which actions are moral/ethical and which are not is referred to as the *principle of double effect* which states it is permissible to indirectly cause harm if the action promotes an even greater good but not to directly cause harm even in pursuit of a greater good. Another explanation of the divergent perceptions to the runaway trolly scenarios is moral. If we consider everyone has equal rights we would be doing something wrong to sacrifice 1 life even if our intent was to save 5 lives. Interestingly, using functional ^18^F-flurodeoxyglucose positron emission tomography (PET) scanning it is possible to identify brain activity in areas concerned with rationality when the decision is whether or not to throw the switch but in areas concerned with emotion involving the decision is whether or not to hurl someone from the bridge. We acknowledge there are other explanations and interpretations of the runaway trolley hypothetical and even that some researcher find it strained at best.

Now apply the *principle of double effect* or morality to possible objections to our modest proposal. Some may argue, without convincing data, that although a transplant in 1st complete remission may harm some people, a greater number of others will gain. They may also argue that recommending a transplant in someone in 1st complete remission is uncertain to harm them as we don’t know if whether that person was cured by chemotherapy or not. However, applying the *principle of double eff*ect or of morality we see we cannot even *potentially* harm someone for a *potential* greater good.

These counter-arguments to our modest proposal also mistake our role as physicians where we make recommendations to individuals, not populations. Our mandate is to recommend the course of action in the best interest of our patient. If we consider someone with AML who has been is in 1st complete remission for a few months, regardless of their prognosis at diagnosis, we must tell them; (1) there is a 20–30% chance they are already cured; (2) there is a 50% chance a transplant in 1st complete remission will not cure them and may accelerate their death or leave them with a complication such as chronic graft-*versu*s-host disease; (3) a 20% chance it may kill them; (4) if they decline a transplant in 1st complete remission and relapse they can potentially receive a transplant; (5) they do not necessarily need to be in a 2nd complete remission to benefit from a transplant; and (6) there are no convincing data a transplant in 1st complete remission will result in a greater likelihood of cure compared with waiting to see if they relapse.

Others might argue data from RCTs can determine if our *modest proposal* is correct. This is another mis-understanding. Although we strongly support using RCTs to gain knowledge this is not our role as someone’s physician. Suppose, for example, a RCT comparing the current practice of transplanting some people with AML in 1st complete remission with our *modest proposal* finds 60% cures (95% Confidence Interval [CI], 50, 70%) *versus* 40% cures (50, 50%) comparing the cohorts. However, the person we are counselling will be cured or not; there is no 60% cure. So the 20% advantage of transplants in 1st complete remission compared with our *modest proposal* is useful were our role to advise a population. This is not applicable when advising an individual. Nor, under the concepts of *principle of double effect* and/or morality (*vide supra*) can we justify recommending a transplant with its attendant risks to someone who might be already cured by chemotherapy. Again, our obligation as physicians is to recommend the best course of action for our patient, not the course of action which will result in greatest good for the greatest number (Hurling someone off the bridge). Interestingly, a recent RCT reported no survival advantage for transplants in 1st complete remission compared with waiting for relapse but awaiting relapse has the advantage of not transplanting anyone already cured by chemotherapy [16]. Other RCTs are in progress (NCT04822766). However, our *modest proposal* is deductive and cannot be proved or refuted to be a wrong strategy at the individual level by data from RCTs studying the performance of cohorts. Patients in 1st complete remission may prefer one or other strategy we discuss. However, there are considerable data patients are swayed by what they perceive as physicians’ preferences, stated or perceived. Elsewhere we and others suggest using scenario planning rather citing statistics is a better way to present therapy options to people [19].

The challenge of how to prevent harming persons with AML in 1st complete remission already cured by chemotherapy is not new. For example, Applebaum and colleagues reported outcomes in a small observational database of subjects with recurrent AML. They found no advantage for transplants in people receiving subsequent chemotherapy whether or not they achieved a 2nd complete remission compared with a transplant done immediately [20]. Importantly, their dataset did not consider people who never advanced to a transplant because of receiving chemotherapy making the argument for immediate transplantation after relapse even more tenable. These authours also suggested waiting for relapse was a reasonable strategy in persons with AML in 1st complete remission whilst discussing limitations of their dataset to answer this question. There are other discussions of this issue but most rely on predicting risk, not preventing harm [21]. Interestingly, current practice has drifted from these considerations with most expert consensus statements and clinical practice guidelines recommending a transplant in 1st complete remission for persons with high- and some with intermediate-risk AML in 1st complete remission [22]. Also, many, if not most, centres recommend trying to achieve a 2nd complete remission before advancing to a transplant and many centres decline transplanting people not in 2nd complete remission. Although these recommendations may be evidence-based at the population level they ignore the mandate to cause no harm to an individual.

Our *modest proposal* focuses on AML in 1st complete remission but, *sensu lato*, it applies to other haematological and solid cancers where a substantial proportion of people may be cured by their initial therapy or are unlikely to progress but where subsequent therapies, some with considerable risks, are given to many. As an example, in myelofibrosis should everyone with low-risk Dynamic International Staging System (DIPSS) score and *JAK2*^V617F^ and *ASXL1* mutations receive a transplant or await possible progression to a higher-risk score. In solid cancers should everyone with stage-2 colo-rectal cancer after definitive surgery receive adjuvant chemotherapy or only those relapsing?. There are no definitive data the latter approach results in worse survival. Or should everyone with stage-2 lung adenocarcinoma with an exon 19 epidermal growth factor receptor (EGFR) mutation receive osimertinib after definitive surgery and chemotherapy or just those relapsing? Again, there are no data there delaying osimertinib results in worse survival. Others have also considered these issues [23].

So, what is our *modest proposal*? Simply put, don’t transplant anyone in 1st complete remission and you will not actively harm someone already cured by chemotherapy. Save transplants for people who relapse and advance immediately to a transplant without attempting to achieve a 2nd complete remission whenever possible.

Our *modest propo*sal differs from recommendations from many clinical practice guidelines and expert consensus panels. For example, Summing up, the 2022 ELN AML recommendations state: *Allogeneic HCT should be considered when the relapse probability without the procedure is predicted to be 35 to 40%*. To support this recommendation the ELN authours cite a 2016 article in *BLOOD* [24] However, the article contains no conceptual-, scientific-, statistical or evidence-based data supporting this recommendation. There are other relevant publications [25].

Although we hope our *modest proposal* will be a tocsin we acknowledge we are not Panglossian and accept it is more likely to be regarded as blasphemous. But, as George Bernard Shaw remarked: *All great truths begin as blasphemies* so we are not discouraged [26] And, like Jonathan Swift, we hold little hope the transplant publik will adopt our *modest proposal*. But we feel compelled to try for consentience from our colleagues acknowledging, as Samuel Butler did: *He that complies against his Will, Is of his own Opinion still, Which he may adhere to, yet disown, For Reasons to himself best known* [27].
